# Host-Defense Activities of Cyclotides

**DOI:** 10.3390/toxins4020139

**Published:** 2012-02-15

**Authors:** David J. Craik

**Affiliations:** Institute for Molecular Bioscience, The University of Queensland, Brisbane, QLD 4072, Australia; Email: d.craik@imb.uq.edu.au; Tel.: +61-7-3346-2019; Fax: +61-7-3346-2101

**Keywords:** circular protein, cyclic peptide, cyclotide, cystine knot, insecticide, kalata B1

## Abstract

Cyclotides are plant mini-proteins whose natural function is thought to be to protect plants from pest or pathogens, particularly insect pests. They are approximately 30 amino acids in size and are characterized by a cyclic peptide backbone and a cystine knot arrangement of three conserved disulfide bonds. This article provides an overview of the reported pesticidal or toxic activities of cyclotides, discusses a possible common mechanism of action involving disruption of biological membranes in pest species, and describes methods that can be used to produce cyclotides for potential applications as novel pesticidal agents.

## 1. Introduction

Cyclotides [1] are a plant-derived family of small proteins characterized by their head-to-tail cyclic backbone and a cystine knot arrangement of three conserved disulfide bonds. They were first discovered in plants from the Rubiaceae (coffee) and Violaceae (violet) families but have since been reported in a range of other plants from the Cucurbitaceae (cucurbit) and Fabaceae (legume) families and it has been predicted that they are widely distributed within the plant kingdom [2]. Cyclotides are notable for their exceptional stability and their diverse range of bioactivities, as well as for their expression in a wide range of plant tissues, including leaves, stems, flowers, dormant seeds and roots [3]. There have been a number of recent reviews on the discovery [4,5,6], structures [4,7,8], and applications [9,10,11,12] of cyclotides, to which the reader is referred for more background but here the focus is on their pesticidal and/or toxic activities.

**Figure 1:**
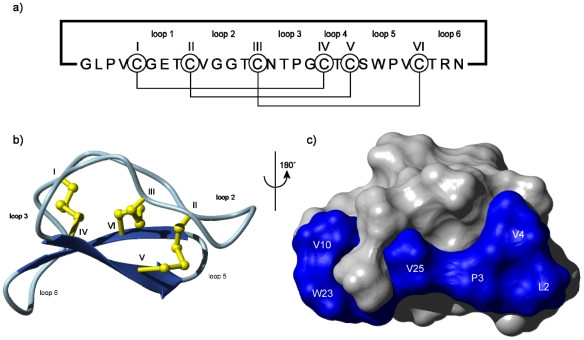
Schematic illustration of (**a**) the sequence and (**b**) the structure of the prototypical cyclotide, kalata B1 showing the head-to-tail cyclic backbone and six conserved cysteine residues (numbered using Roman numerals) that are connected together in a I–IV, II–V and III–VI connectivity leading to a cystine knot [15]. This connectivity has been established by a range of chemical and NMR methods [16,17]. The structure folds up into a compact three-dimensional shape that contains a β-sheet motif (indicated by the broad arrows) as well as a series of turns. The backbone segments between cysteine residues are referred to as loops and are numbered 1–6. Some loops, e.g. loops 1 and 4, are relatively highly conserved, whereas others are typically hypervariable in cyclotides from different plants, leading to the description of cyclotides as a natural combinatorial template [18]; (**c**) Space-filling view of the surface of kalata B1 showing the location of a surface-exposed patch of hydrophobic residues.

The structure of the prototypical cyclotide, kalata B1, is shown in Figure 1 [13], highlighting that cyclotides are around 30 amino acids in size with a cyclic peptide backbone. They fold into compact three-dimensional shapes that incorporate a small β-sheet structure and a series of turns built around the cystine knot core. This compact structure and elaborate cross-bracing by disulfide bonds is a main contributing factor that makes cyclotides exceptionally stable. They are highly resistant to chemical, thermal and enzymatic treatments that would typically destroy conventional proteins [14].

Individual plants express a suite of cyclotides, sometimes comprising more than 100 different sequences. Based on the reported discovery rates for cyclotides in the Violaceae and Rubiaceae and the large number of unique cyclotides per plant species it has been predicted that there might be ~50,000 cyclotide sequences in nature [2] but this is probably an underestimate based on recent discoveries in the Fabaceae [19,20]. Table 1 shows a representative selection of cyclotides from the three major subfamilies that have so far been identified. The Möbius and bracelet subfamilies are the largest subfamilies of cyclotides and are so named because of the presence or absence of a conceptual 180° twist in the peptide backbone brought about by a cis X-Pro peptide bond in the Möbius class and the lack of this “twist” in the circular ribbon of the bracelet class [1]. A smaller subfamily of cyclotides known as the trypsin inhibitor cyclotides has quite different sequences than the other two subfamilies and is also routinely classified as “cyclic knottins” based on their sequence homology to conventional (acyclic) cystine knot proteins from the knottin family [21]. The cyclotides shown in Table 1 have been structurally characterized and their relevant PDB ID codes are given in the table, along with their sequences and some biophysical characteristics, including net charge and size (numbers of amino acids).

**Table 1:** Representative cyclotides from the Möbius, bracelet and trypsin inhibitor subfamilies.

Cyclotide ^a^	AA ^b^	Ch ^c^	Sequence ^d^	PDB ^e^	Ref.
**Möbius subfamily**					
kalata B1	30	+2	GLPVCG**E**TCVGGTCNTPGCTCSWPVCTRN	1NB1	[13]
kalata B2	29	−1	GLPVCG**E**TCFGGTCNTPGCSCTWPICTRD	1PT4	[22,23]
kalata B7	29	+1	GLPVCG**E**TCTLGTCYTQGCTCSWPICKRN	2JWM	[24]
kalata B12	28	−2	GSLCG**D**TCFVLGCNDSSCSCNYPICVKD	2KVX	[25]
cycloviolacin O14	31	+3	GSIPACG**E**SCFKGKCYTPGCSCSKYPLCAKN	2GJ0	[26]
varv F	29	0	GVPICG**E**TCTLGTCYTAGCSCSWPVCTRN	3E4H	[27]
**Bracelet subfamily**					
circulin A	30	+2	GIPCG**E**SCVWIPCISAALGCSCKNKVCYRN	1BH4	[28,29]
circulin B	31	+2	GVIPCG**E**SCVFIPCISTLLGCSCKNKVCYRN	2ERI	[29]
cycloviolacin O1	30	0	GIPCA**E**SCVYIPCTVTALLGCSCSNRVCYN	1NBJ	[26]
cycloviolacin O2	30	+2	GIPCG**E**SCVWIPCISSAIGCSCKSKVCYRN	2KNM	[26]
kalata B5	30	−1	GTPCG**E**SCVYIPCISGVIGCSCTDKVCYLN	2KUX	[30,31]
kalata B8	31	+1	GSVLNCG**E**TCLLGTCYTTGCTCNKYRVCTKD	2B38	[32]
tricyclon A	33	−1	GGTIFDCG**E**SCFLGTCYTKGCSCGEWKLCYGTN	1YP8	[33]
palicourein	37	−1	GDPTFCG**E**TCRVIPVCTYSAALGCTCDDRSDGLCKRN	1R1F	[34,35]
vhl-1	31	0	SISCG**E**SCAMISFCFTEVIGCSCKNKVCYLN	1ZA8	[36]
vhl-2	30	−1	GLPVCG**E**TCFTGTCYTNGCTCDPWPVCTRN	2KUK	[36,37]
vhr1	30	0	GIPCA**E**SCVWIPCTVTALLGCSCSNKVCYN	1VB8	[3]
**Trypsin inhibitor subfamily**					
MCoTI-II	34	+3	GGVCPKILKKCRRDSDCPGACICRGNGYCGSGSD	1IB9	[38,39]

^a^ Selected examples only are shown. For a full listing of cyclotides see CyBase [40]; ^b^ AA refers to the number of amino acids in the sequence; ^c^ Ch refers to the net charge of the cyclotide; ^d^ shows the sequences using one-letter amino acid codes, with the conserved Glu (E) bolded; ^e^ PDB ID code for 3D coordinates.

Cyclotides were originally discovered based on their observed bioactivities. The prototypical member of the family, kalata B1, was identified as the active uterotonic agent in a medicinal tea used by women in Africa to accelerate child birth. The plant used to make the tea is *Oldenlandia affinis*, a member of the Rubiaceae family of plants [41]. The first cyclotide discovered from the violet family, violapeptide 1, was fortuitously discovered while looking for haemolytic activity of saponins [42]. In one of the most significant early reports, a series of circular peptides was discovered in a screening program searching for anti-HIV molecules [29,43,44]. Similarly, cyclopsychotride A [45] was discovered during a screen for neurotensin antagonists. These initial bioassay-guided discoveries led to systematic searches for cyclotides based on their structural properties and this eventually led to the discovery of a wide range of other activities for cyclotides, including antimicrobial [46], insecticidal [22,47] and other pesticidal activities. I will describe these biological activities in more detail in Section 3, after making some brief comments on the biosynthesis and artificial synthesis of cyclotides.

**Figure 2:**
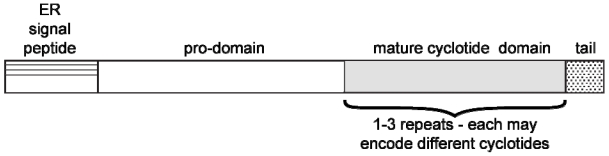
Schematic illustration of the architecture of cyclotide precursor proteins from the Violaceae and Rubiaceae. They comprise an ER signal sequence, an *N*-terminal prodomain, the mature peptide sequence and a *C*-terminal pro-peptide. Some precursors contain multiple copies (*i.e.*, up to three copies) of the cyclotide domain flanked by short pro-peptide regions. Recent studies of cyclotides from the Fabaceae family have suggested that they are produced from what appears to be an ancestral albumin gene [20,52], suggesting that plants have evolved several alternative mechanisms for the biosynthesis of cyclotides.

Cyclotides are biosynthesized ribosomally as precursor proteins that encode one or more cyclotide domains [48,49]. Figure 2 shows the generic arrangement of a typical cyclotide precursor protein, which comprises an endoplasmic reticulum (ER) signal sequence, a pro-domain, a mature cyclotide domain and a *C*-terminal region. Although the excision and cyclization processes that yield cyclic mature peptides from these precursors are not fully understood, it is believed that asparaginyl endoproteinase (AEP) enzyme activity plays an important role [50,51]. This hypothesis is consistent with the presence of an absolutely conserved Asn (or Asp) residue that at the *C*-terminus of the cyclotide domain within the precursor proteins. (e.g., see cyclotide sequences in Table 1). It is further supported by studies involving expression of mutated cyclotides in transgenic plants that have identified residues important for processing. For example mutation of the conserved Asn to Ala abolishes production of cyclic peptides *in planta* [51].

## 2. Pesticidal and/or Toxic Activities

The natural function of cyclotides is thought to be for the protection of plants against pests, a suggestion prompted by the discovery that cyclotides potently inhibit the growth and development of certain insect larvae [47]. It is not known why an individual plant species can express so many different cyclotides. This phenomenon might have evolved to counter the development of resistance of pests to an individual defense molecule, or it might reflect that fact that cyclotides have an array of functions in plants that have so far not been identified. For example, some host defense peptides in animals have direct antimicrobial activity but also function as signaling molecules and immune modulators. Similar multifunctional roles might be possible for cyclotides but have so far not been reported. In this article the focus is on toxic activities of cyclotides. Table 2 summarizes examples of pesticidal and/or toxic activities of cyclotides. They include hemolytic, cytotoxic, antimicrobial, insecticidal, antifouling, molluscicidal and nematocidal activities.

**Table 2:** Pesticidal and/or toxic activities reported for selected cyclotides.

Cyclotide	Activity	Ref.
***Möbius subfamily***		
kalata B1	Insecticidal, molluscicidal, hemolytic, nematocidal, antibacterial, anti-HIV	[47,53,54,55,56]
kalata B2	Insecticidal, molluscicidal, nematocidal, antibacterial	[22,56,57]
kalata B5	Molluscicidal	[56]
cycloviolacin O14	Nematocidal, anti-HIV, hemolytic	[26]
varv A & F	Cytotoxic	[27,58]
***Bracelet subfamily***		
circulin A & B	Hemolytic, anti-bacterial, anti-HIV	[29]
cycloviolacin O1	Nematocidal, molluscicidal	[26,56,59,60]
cycloviolacin O2	Nematocidal, cytotoxicity, hemolytic, anti-barnacle, antibacterial	[57,61]
Cycloviolacin Y1	Hemolytic, anti-HIV	[62]
vhl-1	Nematocidal, anti-HIV	[36]
***Trypsin inhibitor subfamily***		
MCoTI-II	Protease inhibition	[63,64,65]

### 2.1. Hemolytic and Cytotoxic Activities

Hemolytic activity, *i.e.*, the ability to cause lysis of erythrocytes, was one of the first activities reported for cyclotides and indeed led to the discovery of violapeptide-I [42]. Overall, cyclotides are only mildly hemolytic, with median hemolytic doses (HD_50_ values) of 10–1000 μM [66] which is weak compared to the potent hemolytic agent melittin from bee venom with a HD_50_ of ~1 μM. Interestingly, hemolytic activity is lost on linearization of cyclotides [67], showing that the intact cyclic backbone is important for this activity, as also appears to be the case for a number of other cyclotide activities, including anti-HIV activity.

So far there have been no reports describing a functional role for this hemolytic activity in a specific host defense interaction. Most investigations of the hemolytic activity of cyclotides have been more concerned with engineering out this activity for cases where a cyclotide framework is targeted for use as a pharmaceutical template. For example, an alanine scan identified a region of the kalata B1 surface in which substitution of any one of nine resides with Ala significantly reduced the hemolytic activity [68].

Various cyclotides have been reported to be cytotoxic to other human cell lines aside from erythrocytes. Cycloviolacin O2, a bracelet cyclotide, has been a particular focus of these studies and of most interest is a small degree of selective toxicity to cancer cell lines relative to normal cells, opening the possibility of uses of cyclotides in anti-cancer activity [58,69,70,71]. This activity however did not hold-up to be useful in a mouse tumor model [72]. The reasons for a lack of *in vivo* activity are not fully understood, but could include high clearance rates or poor distribution to the site of action in a poorly vascularized tumor model [72]. Also of concern from a pharmaceutical perspective is the finding that cycloviolacin O2 has a very abrupt toxicity profile, with lethality in mice at a single injection of 2 mg/kg but no signs of discomfort to the animals at 1.5 mg/kg [72].

Overall the cytotoxic activities of cyclotides appear to be confined to members of the Möbius and bracelet subfamilies and toxic activities have not been reported for members of the trypsin inhibitor subfamily.

### 2.2. Antimicrobial Activity

Antimicrobial activity of several synthetically produced cyclotides was first reported in 1999 [46] and there have only been a few other primary reports of this activity since then [20,73,74]. Overall, it appears that not all cyclotides are antimicrobial and that the positive examples have activity typically only under low salt conditions. In the initial study [46] four synthetically produced cyclotides, kalata B1, circulin A, circulin B and cyclopsychotride A, were tested against a range of human pathogenic bacteria, including *E. coli* and *Staphylococcus aureus*. The most potent example was circulin A against *S. aureus*, which had a minimum inhibitory concentration (MIC) of 0.19 µM, but kalata B1 was also reported to be highly potent (MIC = 0.26 µM) against *S. aureus*. Kalata B1 was inactive against *E.coli* under either high or low salt conditions but a later study reported conflicting data in *E.coli*. [73] Recent studies have confirmed antimicrobial activity for cycloviolacin O2, [74] and hedyotide B1 [20]. These studies have focused on testing against human pathogenic bacteria and it appears that there have been no systematic studies of the effect of cyclotides on plant-pathogenic bacteria. A recent report [57] did however examine the toxic effects of cyclotides against soil bacteria, and against several plants and algae, to determine the environmental effects of cyclotides, as further described in Section 6.

### 2.3. Insecticidal Activity

The first report of insecticidal activities of cyclotides was in 2001 where kalata B1 was shown to inhibit the growth and development and increase the mortality of Helicoverpa larvae presented with an artificial diet containing cyclotides [47]. When present in a diet at approximately the same concentration as it occurs in natural leaf tissue (~0.8 µmol/g), kalata B1 was found to be a potent insecticidal agent. These findings have since been reproduced in studies of kalata B2 [22] from *O. affinis* and Cter M from *Clitoria ternatea*. [19]. The mechanism of the insecticidal activity appears to be by disruption of the mid-gut membranes of larvae that have ingested cyclotides, as judged from electron microscopy studies [75]. Although insecticidal activity is presumed to be the primary natural biological function of cyclotides [76] there have not yet been systematic reports of their testing against a range of insect species, nor have there been studies of possible synergistic effects of cyclotides, given that a single plant often expresses a large number of cyclotides in any one tissue [12].

### 2.4. Anti-Fouling Activities

Cycloviolacin O2 has been reported to have activity against the barnacle *Balanus improvisus* in the sub-micromolar range [61]. Although there potentially could be applications of this activity in defouling ship hulls, the cost of cyclotides is likely to be a limiting factor in precluding such applications, unless considerably cheaper production methods for cyclotides can be developed than are currently available. It is interesting that cycloviolacin O2 is a member of the bracelet subfamily of cyclotides and that in general this subfamily tends to be more active in toxic properties against many organisms relative to the Möbius subclass.

### 2.5. Molluscicidal Activities

Cyclotides tested against *Pomacea canaliculata,* the Golden Apple snail, a major pest of rice in South East Asia have shown promising activities. This snail was originally imported into Taiwan from South America in the 1980s and has now spread widely in agricultural wetlands in Japan, the Philippines and Taiwan where it has caused crop damage estimated to have cost billions of dollars. The cyclotides cycloviolacin O1, kalata B1 and kalata B2 were found to be more toxic to *Pomacea canaliculata* than the commercially used molluscicide metaldehyde, whereas kalata B7 and B8 were significantly less toxic [56]. These assays showed that there was a dose dependent effect on snail mortality when cyclotides were introduced into the aquatic environment of the snails. The LC_50_ (median lethal concentration) was 53 µM for kalata B2 and 133 µM for metaldehyde. Tests of kalata B2 against a non-target fish species, *Oreochromis niloticus*, which has been used as a biological control agent in rice fields, revealed toxicity lower than rotenone, a naturally occurring commercial piscicidal agent. However, further detailed investigations on the relative toxicities of cyclotides against target and non-target species are required before cyclotides could be used to develop natural molluscicides.

### 2.6. Nematocidal Activities

The nematocidal activity of cyclotides has been extensively studied over the last few years, particularly against livestock pest nematodes [55,59,60]. In the first study [55] the *in vitro* effects of kalata B1, B2, B3, B5, B6 and B7 on the viability of egg, larval, and adult life stages of two species of economically important gastrointestinal nematode parasites of livestock, *Hemonchus contortus *and *Trichostrongylus colubriformis* were examined. The cyclotides showed significant activity in inhibiting development of nematode larvae and motility of adult worms, with activities comparable to some commercially used anthelmintic compounds. For example, kalata B6 was the most potent of the natural cyclotides tested, having a larvicidal activity (IC_50_) of 2.6 µg/mL against *H. contortus*. [55]. Alanine mutants of kalata B1 were assayed against larvae to determine residues responsible for activity and it was found that the anthelmintic activity was dramatically reduced as a consequence of the mutation of a series of residues clustered on one face of the molecule. Activities toward larvae were equivalent in the naturally occurring L-isomer of kalata B1 and a synthetic all-D-isomer, indicating that there is no chiral requirement for anthelmintic activity [55]. The clustering of “bioactive” residues and the lack of chiral selectivity supported a proposed mode of action of cyclotides that involves a membrane-based interaction rather than interaction with a stereospecific receptor. The cyclotide-induced leakage of a fluorescent dye from vesicles used as a model membrane mimetic further confirmed the membrane lytic ability of cyclotides. The relative potency of kalata B1 and kalata B2 in causing membrane leakage was consistent with the order of their anthelmintic activity.

This initial study was followed by a series of investigations that led to improved anthelmintic activity against these livestock pests [59,77], and also demonstrated activity against canine and human hookworms [60]. Overall, the results demonstrate that the cyclotides show potential for use in the control of nematode parasites of both agricultural and medical importance.

## 3. Mechanisms of Action

**Figure 3:**
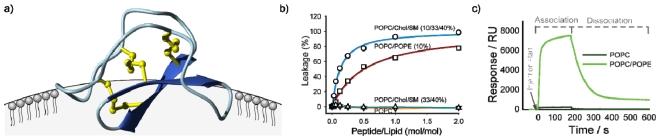
Schematic illustration of studies done to establish membrane binding of cyclotides. Panel (**a**) shows preferred binding mode of kalata B1 to DPC micelles as deduced from NMR studies, indicating binding via the hydrophobic patch [78,80]; (**b**) shows vesicle leakage studies in which the addition of cyclotides to phospholipid vesicles causes leakage of vesicle contents in a dose dependent manner, with differential effects for different lipid compositions made up of palmitoyloleoylethanolamine (POPC), palmitoyloleoylethanolamine (POPE), cholesterol (Chol) and sphingomyelin (SM) [83]; (**c**) shows surface plasmon resonance studies indicating preferential binding to PE compared to PC lipids [83].

Although the range of biological activities of cyclotides might seem diverse, in fact they all appear to be accounted for by a common mechanism involving interactions of cyclotides with membranes and their subsequent disruption. In an early biophysical study NMR chemical shifts and diffusion measurements were used to demonstrate a specific interaction between a model membrane in the form of dodecylphosphocholine (DPC) micelles and the cyclotide kalata B1 [78]. This was followed by similar studies on B7 [79], which confirmed that a hydrophobic patch on the surface of the cyclotides (shown in Figure 1c) was the primary interaction face with membranes. The orientation of kalata B1 binding to the DPC surface is shown in Figure 3a. The precise orientation varies with different cyclotides, dependent on the location of the surface-exposed hydrophobic patch [80].

Independent studies showed that the membrane binding was functional, in that cyclotides were able to induce leakage of contents from phospholipid vesicles (Figure 3b) and form large pores in lipid bilayers [81], and that membrane binding modulated cytotoxicity [69]. Surface plasmon resonance studies [82,83] also established a specific interaction between cyclotides and phospholipid bilayers, as shown in Figure 3c [83]. These various studies have provided details on the selectivity of particular cyclotides for particular lipid subtypes. Overall, cyclotides appear to have a preference for phosphatidylethanolamine (PE) as opposed to phosphatidylcholine lipids (PC). Furthermore, for membranes of a given PE content kalata B1 has a higher affinity for membranes in a liquid disordered phase (*i.e.*, more rigid membranes rich in cholesterol (Chol) and sphingomyelin (SM) that are raft-like domains [83]).

The ability to chemically synthesize or modify cyclotides has been important in establishing structure-activity relationships. As noted earlier, chemically modified cyclotide derivatives in which individual residues are successively replaced by alanine established that there was a patch of residues clustered on the surface of cyclotides that is important for biological activity [68]. This bioactive patch is not co-located with the hydrophobic patch implicated in membrane binding, which led to the suggestion that self-association may be involved in the mediation of cyclotide activity. Chemical modification studies have also established the importance of a key glutamic acid in regulating the activity of cyclotides. Esterification of this residue causes a marked drop in biological activity [84]. This residue is at the centre of the bioactive patch of residues on the kalata B1 cyclotide surface. The Glu residue is conserved in all but one of the currently known naturally occurring cyclotides, with the exception being kalata B12, where it is replaced with the conservative Asp residue. However, the precise role of the Glu in determining cyclotide activity is yet to be delineated.

In another example of the value of chemistry in determining structure-activity relationships of cyclotides a recent study used a chemically synthesized all-D kalata molecule to confirm the membrane binding hypothesis [83]. The insecticidal, anti-HIV and hemolytic activity of all-D and all-L mirror image forms of kalata B1 were similar, suggesting that a stereospecific receptor is not involved in the interaction. The slightly lower activity for the all-D peptide can be attributed to the slight chirality associated with membranes.

In summary, although cyclotides have a diverse range of activities, including antimicrobial, anti-HIV and hemolytic, and insecticidal, all these activities can be attributed to membrane binding interactions. In the case of insecticidal activity electron microscopy studies have shown clear disruption to the surface cells in the midgut of Lepidopteran larvae fed on a cyclotide-containing diet [75].

## 4. Methods of Production of Cyclotides

**Figure 4:**
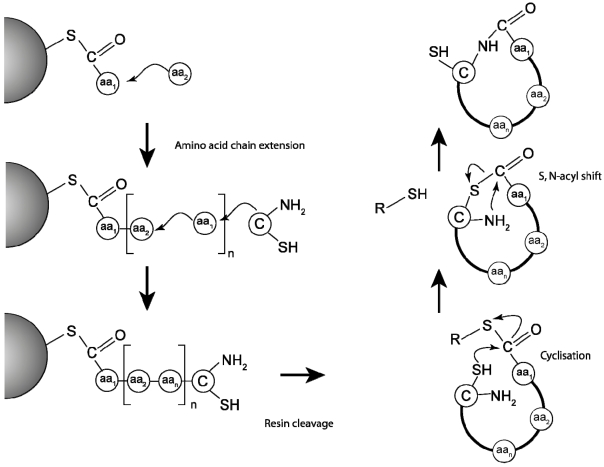
Schematic illustration of synthetic chemical approach for the production of cyclotides. The peptide chain is assembled using solid phase peptide chemistry (SPPS) with the *C*-terminal residue (aa_1_) linked to the resin using a thioester linker. Amino acids are added sequentially and the chain completed with an *N*-terminal cysteine. Since cyclotides contain six cysteines there are thus six possible linear cyclotide designs that could be used to produce a given cyclotide [87]. The linear cyclotide is cleaved from the solid-phase resin under acidic conditions. Cyclization occurs under basic conditions through a trans-esterification reaction and spontaneous S, *N*-acyl shift.

To commercially exploit cyclotides as pesticidal agents it will be necessary to develop cost-effective approaches for delivering them to target plants. Synthetic methods to produce cyclotides have been developed over the last decade, and include solid phase peptide synthesis approaches [46,85,86,87,88] as well as chemo-enzymatic [64,65,89,90] and biological approaches involving modified inteins [91,92,93]. So far, most of the proof-of-concept work on the structure-activity relationships of cyclotides has been done using chemically synthesized cyclotides analogues. Solid phase chemistry is the preferred method here and typically the ligation of the *N* and the *C* terminus is achieved using an adaptation of “native chemical ligation” chemistry [94,95]. This requires that the peptide chain be assembled linked to the resin with a thioester and the linear precursor sequence assembled in such an order that the *N*-terminal residue is a cysteine. This allows a trans-thioesterification reaction to achieve the cyclization, as highlighted in Figure 4.

This technology works well for Möbius trypsin inhibitor subfamily cyclotides although in the past the folding of the bracelet cyclotides has been problematic [96]. Several approaches have recently been developed to overcome this limitation and further improvement seems likely in the future [97,98].

Another approach to making cyclotides is to assemble them as conventional linear peptides and then achieve the cyclization enzymatically. This chemo-enzymatic approach can be done by engineering, for example, a trypsin active site into the linear cyclotide precursor and using trypsin to achieve the ligation. Proof-of-concept for this approach was established early for the cyclic peptide SFTI-I [99] and then adapted to the trypsin inhibitor cyclotide MCoTI-II [64,65,89,90]. One advantage of this approach is that the enzyme can be immobilized on a column and the cyclization achieved while the substrate flows through the column, thus leading to easy purification.

Biological approaches to cyclotide synthesis have also been developed using *E. coli* as the host cells and modified inteins for activation of linear cyclotide precursors. This approach has been demonstrated for a range of cyclotides, including kalata B1 and MCoTI-II and has been used to produce small libraries of cyclotides and other cyclic peptides [11,91,92,93,100,101]. Although at this stage the yields are still relatively low compared to solid phase peptide synthesis approaches the biological approach has the potential to be less expensive.

In another biological approach, significant progress has been made in using plant cell culture methods for the production of native cyclotides. Specifically, Dörnenburg *et al*. [102,103,104,105] have developed a plant cell fermentation system and demonstrated excellent yields of native cyclotides in cultured *O. affinis* cells. Interestingly, the cultured cells not only produced cyclotides that had been identified in whole plant extracts of *O. affinis* but also produced some novel cyclotides not previously seen.

Despite the promise seen with SPPS or whole-cell-based approaches to cyclotide production, it seems likely that for large scale pesticidal applications of cyclotides a transgenic approach in which cyclotide genes are inserted directly into target plants is likely to be the most cost effective method. The main advantage of this approach is that the cyclotides are expressed only in the target organism thus potentially minimizing environmental impacts seen with traditional pesticidal applications through spraying. Proof-of-concept that cyclotides can be produced in model plants (tobacco and Arabidopsis) has been obtained in studies aimed at understanding the mechanistic basis of cyclotide processing [50,51].

## 5. Safety and Breakdown

The potential applications of cyclotides as pesticidal and/or pharmaceutical agents, merit safely evaluations of their effects in the environment. In a recent study [57] it was found that cyclotides were toxic to a range of test organisms, with EC_50_ values ranging from 12 to 140 µM against algae, 9–40 µM against duckweed, 4–50 µM against lettuce and 7–26 µM against soil bacteria. It was concluded that cyclotides might adversely affect solid and aquatic environments and that this should be taken into account in future risk assessments for cropping systems producing cyclotides. However, it also needs to be noted that cyclotides appear to be widely present naturally in a wide range of plants from the Rubiaceae, Violaceae, Cucurbitaceae and Fabaceae families and there are so far no reports of toxic effects in the environments associated with these naturally occurring cyclotides.

There have only been two studies that have reported chemical breakdown products of cyclotides [31,84] and it seems that more work in understanding cyclotide turnover is warranted to assess how stable these molecules are in their natural environment.

## 6. Conclusions

Although cyclotides were discovered only 15 years ago, they have developed into an exciting field of research and display a wide range of biological activities. At this stage it would appear that these activities are all associated with membrane interactions and given a demonstrated preference for cyclotides for some phospholipids over others it seems likely that cyclotides are targeted to specific cell types based on membrane composition to exert their biological activity. The rate of cyclotides discovery is rapidly evolving and the prediction of at around 50,000 members of the family may turn out to be an underestimate. Significant progress has been made in the chemistry for synthesizing cyclotides as well as in their biological synthesis. Thus there are now available an array of tools for the chemical production and modification of cyclotides, allowing further refinement of structure-activity relationships. Significant progress has also been made in the production of transgenic plants expressing cyclotides and so there appears to be great potential for the use of cyclotides as pesticidal agents, and indeed for modified cyclotides as medicines.
